# Vaginal Microbiota and Mucosal Pharmacokinetics of Tenofovir in Healthy Women Using a 90-Day Tenofovir/Levonorgestrel Vaginal Ring

**DOI:** 10.3389/fcimb.2022.799501

**Published:** 2022-03-08

**Authors:** Andrea R. Thurman, Jacques Ravel, Pawel Gajer, Mark A. Marzinke, Louise A. Ouattara, Terry Jacot, M. Melissa Peet, Meredith R. Clark, Gustavo F. Doncel

**Affiliations:** ^1^ Department of Obstetrics and Gynecology, CONRAD, Eastern Virginia Medical School, Norfolk, VA, United States; ^2^ Institute for Genome Sciences and Department of Microbiology and Immunology, University of Maryland School of Medicine, Baltimore, MD, United States; ^3^ Departments of Pathology and Medicine, Johns Hopkins University School of Medicine, Baltimore, MD, United States

**Keywords:** vaginal microbiome, tenofovir diphosphate, PrEP, multipurpose prevention technology, pre-exposure (PrEP) prophylaxis

## Abstract

**Background:**

A relationship between the vaginal microbiota and tenofovir (TFV) concentrations and activity after topical administration has been previously reported.

**Objective:**

CONRAD A15-138 was a randomized, placebo-controlled Phase I study aimed at characterizing the safety, pharmacokinetics (PK), and pharmacodynamics (PD) of TFV and levonorgestrel (LNG) administered through a vaginal ring (IVR) for 90 days. Herein, we describe changes from baseline in the vaginal microbiota with IVR use and the impact of the vaginal microbiota on mucosal TFV PK.

**Methods:**

The study screened 68 participants and randomized 47 (37 TFV/LNG, 10 placebo), assessing the vaginal microbiota by sequencing the V3–V4 regions of 16S rRNA genes prior to IVR insertion and monthly for 3 months. Concentrations of TFV in vaginal fluid (VF), and TFV and TFV-diphosphate (TFV-DP) in vaginal tissue, and modeled PD against HIV-1 *in vitro* were measured before and after treatment.

**Results:**

There were no clinically significant changes in relative abundance of vaginal bacterial phylotypes from pre-insertion baseline at any month among active and placebo IVR users. There were no significant changes in community state type (CST) with IVR use. Participants with diverse, anaerobic CST IVA/B microbiota had higher *in vivo* release of TFV from the IVR compared to women with *Lactobacillus*-dominated (LbD) microbiota, who had expected *in vivo* TFV release rates. Median VF TFV concentrations were significantly higher among women with CST IVA/B microbiota in months 1 (3,135 ng/mg VF) and 2 (3,800 ng/mg). Women with LbD microbiota had significantly higher median VF TFV concentration (1,423 ng/mg) and median TFV (103 ng/mg) and TFV-DP (5,877 fmol/mg) tissue concentrations versus women with CST IVA/B microbiota at month 3. All women demonstrated a significant increase from pre-insertion baseline of *in vitro* HIV-1 inhibition by VF (p values <0.05). PD differences in tissue according to CST, however, were not statistically significant.

**Conclusion:**

TFV/LNG IVR use did not change the vaginal microbiota nor increase the incidence of CST IVA/B. Vaginal microbiota, and in particular CST IVA/B, possibly through increased vaginal pH, impacted *in vivo* TFV release and cervicovaginal (CV) PK, but both PK and PD data suggest CV protection against HIV-1.

**Clinical Trial Registration:**

ClinicalTrials.gov (#NCT03279120)

## Introduction

Products that offer protection against multiple sexually transmitted infections (STIs) [e.g., herpes simplex virus type 2 (HSV-2) and human immunodeficiency virus type 1 (HIV-1)], or both STIs and unintended pregnancy, termed multipurpose prevention technologies (MPTs), are urgently needed to improve global health. Women have overwhelmingly indicated that they prefer an MPT product over single-indication prevention products (El-Sahn et al., 2018). CONRAD and our collaborators developed two MPT intravaginal rings (IVRs) which release tenofovir (TFV) alone (active against HIV-1 and HSV-2) and TFV in combination with levonorgestrel (LNG) (contraceptive) for 90 days (Johnson et al., 2012; Clark et al., 2014). To date, the TFV and or TFV/LNG IVRs have been tested in 5 phase I studies (CONRAD 128 ClinicalTrials.gov NCT02235662 (Thurman et al., 2018; Thurman et al., 2019), CONRAD 138 NCT03279120 (Thurman et al., 2021), CONRAD 140 NCT02722343 (Ouattara et al., 2022a), CONRAD 144 NCT03762382 (Mugo et al., 2021), and MTN038 NCT03670355, conducted in the United States, Dominican Republic, and Western Kenya.

Bacterial vaginosis (BV) is characterized by a vaginal microbiota comprising a diverse set of strict and facultative anaerobes and a lack of protective *Lactobacillus* species. BV is the most common cause of vaginal discharge worldwide (Johnson et al., 2005; Kenyon et al., 2013). Approximately 85% of women with BV are asymptomatic (Koumans et al., 2007). BV is associated with an increased risk of several adverse reproductive health outcomes including preterm labor (Sanu and Lamont, 2011) and STI (Wiesenfeld et al., 2003; Cherpes et al., 2003; Gallo et al., 2012; Brotman et al., 2012; Balkus et al., 2014; Mitra et al., 2016), including HIV-1 acquisition (Taha et al., 1998; Myer et al., 2005; Atashili et al., 2008) [reviewed in (Thurman and Doncel, 2011)]. Therefore, assessment of the vaginal microbiota is part of the safety evaluation of HIV-1 prevention products, particularly topical vaginal products.

Recent subset analyses of women using topical, peri-coitally dosed TFV 1% vaginal gel in the CAPRISA 004 cohort and TFV vaginal film from the FAME 04 cohort found that women with vaginal microbiota lacking *Lactobacillus* species had reduced mucosal concentrations of TFV and the active metabolite, TFV-diphosphate (TFV-DP), potentially reducing the efficacy of these peri-coital, topical microbicides (Hillier et al., 2017; Klatt et al., 2017). In the CONRAD 128 study, we previously published that when TFV is delivered continuously for approximately 14 days, *via* an IVR, TFV and TFV-DP concentrations in the cervicovaginal (CV) fluid and vaginal tissues were high, meeting efficacy benchmarks for both HIV-1 and HSV-2 inhibition, whether the woman had optimal *Lactobacillus*-dominated (LbD) microbiota or a non-optimal microbiota characterized by a diverse set of anaerobic bacteria [community state type (CST) IVA/B] (Thurman et al., 2019; McKinnon et al., 2019). We recently completed CONRAD 138, a 90-day study of the TFV/LNG IVR, and reported its main results, showing good safety and tolerability, acceptable bleeding patterns, and benchmark concentrations of TFV and LNG (Thurman et al., 2021). CONRAD 138 differed from the previous CONRAD 128 study in that participants used either a placebo or TFV/LNG IVR cyclically or continuously for the full 90-day duration, rather than 14 days. Through this subanalysis, we characterized the composition of the vaginal microbiota among women using either a placebo or TFV/LNG IVR for up to 90 days and described the association between the vaginal microbiota and mucosal pharmacokinetics (PK) of TFV. We also analyze the relationship between the vaginal microbiota and *in vitro* pharmacodynamic (PD) endpoints before and after IVR use. Given the high prevalence of BV and CST IV-associated vaginal microbiota in adolescent girls and young women in Africa and other high HIV-1 prevalence regions (Johnson et al., 2005; Kenyon et al., 2013; Roxby et al., 2016; Brooks et al., 2017; Achilles et al., 2018), the target end users for these IVRs, the data reported herein are relevant to further development of MPTs.

## Materials and Methods

### Clinical Study

CONRAD A15-138, the ENRICH (Evaluating New Ring Choices) study, was an outpatient, randomized, partially blinded, placebo-controlled, parallel, phase I study conducted at the CONRAD Intramural Clinical Research Center at Eastern Virginia Medical School (EVMS) (Norfolk, VA) and PROFAMILIA (Santo Domingo, Dominican Republic) (Thurman et al., 2021). The study visits and procedures have been previously outlined (Thurman et al., 2021). The sampling relevant to the microbiota investigation is displayed in **Supplementary Table 1**. Briefly, written informed consent was obtained from all participants prior to any study procedures. Participants were healthy, 18–50 years old, had a body mass index (BMI) less than 30 kg/m^2^, and self-reported no use of exogenous hormones and regular menstrual cycles. All women underwent a screening visit to detect the presence of exclusionary factors (e.g., symptomatic BV, active HSV-2*, Neisseria gonorrhoeae*, *Chlamydia trachomatis*, *Trichomonas vaginalis*, HIV-1, Hepatitis B). Volunteers with asymptomatic BV were allowed to enroll in the study. Just prior to IVR insertion, at visit 4 (V4), in the follicular phase (menstrual cycle day 6 ± 1 day), we obtained a vaginal Copan ESwab (Copan Diagnostics, Murrieta, CA, USA) for baseline microbiota analysis. Microbiota samples were initially collected only at baseline and at the end of the 90-day treatment (visit 31, V31). However, we amended the protocol in January 2018 to add the collection of additional samples for vaginal microbiota analysis at the end of month 1 (visit 13, V13) and month 2 (visit 22, V22) of IVR use (**Supplementary Table 1**).

Vaginal microbiota and CV tissue and fluid samplings, although not always collected at the same visit (**Supplementary Table 1**), were performed within an average of 3 days of each other. Vaginal fluid (VF) was sampled with a Dacron swab for the TFV concentration at several times during the study (Thurman et al., 2021), and we selected month 1 (visit 11, V11), month 2 (visit 20, V20), and month 3 (visit 29, V29) VF concentrations to pair with vaginal microbiota samplings (**Supplementary Table 1**). We obtained a CV fluid lavage (CVL) with 4 ml of normal saline at pre-insertion baseline (V4), month 1 (V11), and month 3 (V29) to test the activity of the CVL supernatant against HIV-1_BaL_ *in vitro* (**Supplementary Table 1**).

Ectocervical and vaginal tissue biopsies were obtained with a Tischler forcep for *ex vivo* HIV-1_BaL_ infection prior to IVR insertion in the luteal phase (day 24 ± 2 days) of the menstrual cycle at visit 3 (V3) and at the end of treatment (EOT, V31), for participants enrolled at the EVMS site. We also obtained vaginal tissue for TFV and TFV-DP concentrations at visit 5 (V5) (24, 48, or 72 h after IVR insertion) and at the EOT, V31, for all participants.

### Randomization

At visit 3, eligible participants were randomized to one of 4 study arms (TFV/LNG or Placebo IVR worn continuously for approximately 90 days versus using the assigned IVR in an interrupted, cyclic manner for 3 cycles of 28 days *in vivo* and then 3 days removed with reinsertion). The main rationale for randomizing participants to a continuous versus a cyclic dosing regimen was to determine the impact of the dosing regimen on the menstrual cycle and bleeding patterns reported in the main study manuscript (Thurman et al., 2021). Participants were also randomized to the time of the first post-IVR insertion vaginal tissue PK sampling (24, 48, or 72 h). We utilized a 4:4:1:1 ratio (continuous TFV/LNG IVR:cyclic TFV/LNG IVR:continuous placebo IVR:cyclic placebo IVR). We used electronic randomization within the Medrio electronic data capture system.

The randomization scheme was stratified by site and treatment group to maintain balance within each treatment group with respect to the number of participants randomized to each sampling time point. Trial participants, laboratory staff, investigators, and statistical/data analysts were blinded to study treatment and dosing regimen to the extent possible.

### Study Product

TFV/LNG and placebo IVRs were manufactured under current good manufacturing practices (cGMP) at Particle Sciences (Bethlehem, PA) using manufacturing processes previously described (Johnson et al., 2012; Clark et al., 2014). The unit dose for the IVRs was designed to be approximately 8–10 mg/day of TFV and 20 µg/day of LNG for 90 days of release. The physical characteristics of the active and placebo IVRs were previously reported (Thurman et al., 2018). The IVRs do not require cold chain storage and were stored at room temperature.

### TFV PK Assessment

PK analysis of TFV and the TFV-DP was conducted by the Clinical Pharmacology Analytical Laboratory at the Johns Hopkins University School of Medicine, the same laboratory collaborators for our first-in woman study (Thurman et al., 2018; Thurman et al., 2019). TFV concentrations in VF and vaginal tissue biopsies were determined *via* previously described liquid chromatographic mass spectrometric analyses (Hendrix et al., 2016). Vaginal fluid was collected on Dacron swabs; TFV concentrations were determined from matrix and collection device-specific calibration standards. Assay lower limits of quantitation (LLOQ) were as follows: plasma TFV, 0.31 ng/ml; VF (swab) TFV, 0.625 ng/Dacron swab; vaginal tissue TFV, 0.05 ng/sample. Results were normalized to net swab or weight of tissue analyzed, and TFV concentrations in these matrices were reported as ng/mg. TFV-DP was measured using a previously described indirect approach, in which TFV was quantitated following isolation of TFV-DP from homogenized tissue lysates and enzymatic conversion to the TFV molecule (Bushman et al., 2011). The assay LLOQ for TFV-DP in tissue was 5 fmol/sample, and drug concentrations were normalized to the amount of tissue analyzed (Shieh et al., 2019). All assays were validated in accordance with FDA, Guidance for Industry: Bioanalytical Method Validation recommendations (FDA, 2018).

### TFV PD Assessments

#### Anti-HIV Activity in CVL

CVL was collected prior to IVR insertion, and at months 1 and 3 post use (**Supplementary Table 1**), and an aliquot of the supernatant was frozen at -80°C and batched shipped for evaluation of anti-HIV activity (Beer et al., 2006). After 24 h, cell viability was determined as the proportion of live cells compared to the cell-only control using CellTiter-Glo (Promega Corp. Madison, WI). Anti-HIV activity was similarly determined in the TZM-bl assay by infecting with 3000 TCID_50_ of HIV-1_BaL_ and measuring luminescence after 48 h using Bright-Glo (Promega Corp., Madison, WI). Percent inhibition of HIV-1_Ba-L_ was determined based on deviations from the HIV-1-only control as previously described (Promega Corporation, 2021a; Promega Corporation, 2021b; Wei et al., 2002). Negative HIV-1 percent inhibition values indicates an enhancement of HIV-1 replication *in vitro*, compared to the HIV-1 only control.

#### p24 Antigen Production by Tissue Biopsies Infected *Ex Vivo* With HIV-1_BaL_

One vaginal and one ectocervical biopsy were collected at pre-insertion baseline, and one ectocervical biopsy was collected at the EOT and placed in cryovials filled with chilled RPMI 1640 media (Life Technologies, Carlsbad, CA) containing 10% fetal bovine serum (ATCC, Manassas, VA) and 100 U/ml penicillin and 100 µg/ml streptomycin (Thermo Fisher Scientific, Waltham, MA) (cRPMI). The CV biopsies were exposed to HIV-1_BaL_ (5 × 10^4^ TCID_50_/ml), within 30 min of collection, in the presence of human interleukin-2 (Roche Diagnostics GmbH, Mannheim, Germany) at a final concentration of 100 U/ml. CV biopsies were washed 2–3 h after virus exposure and then cultured in cRPMI media (500 µl) containing IL-2 for 21 days (Ouattara et al., 2022b). We collected the tissue culture supernatant (approximately 300 µl) every 3 to 4 days and replenished cultures with media containing IL-2. We stored the supernatants at -80°C and evaluated them at the end of the culture for HIV-1 p24 antigen expression by ELISA (Perkin Elmer) in pg/mL. Area under the curve (AUC), cumulative (CUM) p24 antigen production, SOFT endpoint (Richardson-Harman et al., 2012), and p24 at Day 21 (D21) were calculated as previously reported (Richardson-Harman et al., 2009).

### Estimation of Residual TFV in TFV/LNG IVRs

Active IVRs were stored in individual sealed foil packages at -80°C until shipped for evaluation of residual drug, as previously reported (Thurman et al., 2021). IVRs were cut at the joint between the LNG segment end cap and the end of the sealed TFV segment to isolate the LNG segment. The LNG segments were then cut into 2–3-mm-thick pieces and dissolved in dichloromethane, polyurethane was precipitated in acetonitrile, and the solution phase was filtered through a 0.2-µm polytetrafluoroethylene filter prior to analysis by high-performance liquid chromatography (HPLC). TFV-containing segments of IVRs were cut into 5–8-mm sections before dissolving residual TFV in 100 ml total volume of sodium phosphate buffer (100 mM, pH 7.4). An aliquot of this solution was further diluted 100-fold before filtration through a 0.2-µm nylon membrane prior to analysis by HPLC. Analysis of TFV and LNG by HPLC was conducted similar to methods described previously (Johnson et al., 2012; Clark et al., 2014). IVR release rates were estimated by subtracting the recovered active pharmaceutical ingredient (API) concentration result from the average control API recovery and dividing by the number of days of reported use.

### DNA Extraction and Vaginal Microbiota Analysis From Vaginal Swabs

Similar to our previous work (Thurman et al., 2019), Copan ESwabs were stored at -80°C at each clinical site and batch shipped on dry ice to the University of Maryland School of Medicine. The swabs were thawed on ice, and 300 µl of the Amies transport medium containing vaginal secretions was processed using the MoBio Microbiome Kit automated on a Hamilton Microlab STAR robotic platform after a bead-beating step on a Qiagen TissueLyser II (20 Hz for 20 min) in 96-deep well plates. Amplification of the V3–V4 regions of the 16S rRNA gene was performed using a two-step PCR as previously described by our group (Holm et al., 2019). Amplicons were visualized on a 2% agarose gel, quantified, and pooled in equimolar concentrations sequenced on an Illumina HiSeq 2500 modified to generate 300-bp paired-end reads (Holm et al., 2019).

The sequences were de-multiplexed using the dual-barcode strategy, a mapping file linking barcode to samples and split_libraries.py, a QIIME-dependent script (Kuczynski et al., 2012). The resulting forward and reverse fastq files were split by sample using the QIIME-dependent script split_sequence_file_on_sample_ids.py, and primer sequences were removed using TagCleaner (version 0.16) (Schmieder et al., 2010). Further processing followed the DADA2 Workflow for Big Data and dada2 (v. 1.5.2) https://benjjneb.github.io/dada2/bigdata.html (Callahan et al., 2016). Forward and reverse reads were each trimmed using lengths of 255 and 225 bp, respectively, filtered to contain no ambiguous bases, minimum quality score of two, and required to contain less than two expected errors based on their quality score. Reads were assembled and chimeras for the combined runs removed as per dada2 protocol. Taxonomic classification was performed using speciateIT (Rosen et al., 2017) and relative abundance reported.

Using VALENCIA, a novel tool that standardizes CST assignments (France et al., 2020), 7 different CSTs were identified from the participant samples (Ravel et al., 2012). The 7 CSTs, defined by the Ravel lab (France et al., 2020), are CST I—*L. crispatus* dominated, CST II—*L. gasseri* dominated, CST III—*L. iners* dominated, and CST V—*L. jensenii* dominated. Three CSTs do not have a high relative abundance of *lactobacilli*; these are CST IV-A, IV-B, and IV-C. CST IV-A has a high relative abundance of *Candidatus Lachnocurva vaginae* [formerly known as BVAB1 (Holm et al., 2020)] and a moderate relative abundance of *G. vaginalis*, while CST IV-B has a high relative abundance of *G. vaginalis* and low relative abundance of *Ca. L. vaginae*. Both IV-A and IV-B have moderate relative abundances of *Atopobium vaginae*. Samples assigned to CST IV-C have a low relative abundance of *Lactobacillus* spp., *G. vaginalis*, *A. vaginae*, and *Ca. L. vaginae* and were instead characterized by the abundance of a diverse array of facultative and strictly anaerobic bacteria. The characterization of the composition of the vaginal microbiota was conducted between April 2019 and August 2019, with data analyses extending to December 2019.

### Ethics and Trial Registration

The study was conducted following the ethical standards outlined in the Helsinki Declaration (1983). The study was approved by the Advarra Institutional Review Board (IRB) (#Pro00022358) for the EVMS site and Comisiòn Nacional de Bioetica (#030-2017) for the PROFAMILIA site. This clinical trial was registered with ClinicalTrials.gov (#NCT03279120) https://clinicaltrials.gov/ct2/results?cond=&term=conrad+138&cntry=&state=&city=&dist.

### Statistical Analyses for Comparing Changes in Vaginal Microbiota Between Baseline and Each Month of Use

In this Phase I clinical study, sample size was based on the study’s primary endpoint of safety and description of mucosal PK rather than statistical considerations for the *post hoc* analysis of the interaction between TFV concentrations and vaginal microbiota. A subject-level comparison of changes in bacterial phylotype relative abundances at months 1, 2, and 3 (EOT) from baseline and pre-IVR insertion in either the active TFV/LNG or placebo treatment groups was calculated by computing the log ratio: Θ = log (relative abundance at visit 13, visit 22, or visit 31/relative abundance at pre-insertion baseline visit 4). The Bayesian hierarchical model was used to estimate if the difference between the mean relative abundances of each bacterial phylotype at month 1, month 2, and month 3 (Θm1, Θm2, Θm3, respectively), compared to mean relative abundances of each bacterial phylotype at pre-insertion baseline (ΘBL), was significantly different from 0; p values and false discovery q values are reported. Further categorization was done based on continuous or cyclic dosing groups for each IVR.

CST transitions between baseline, pre-IVR insertion, and months 1, 2, and 3 were described for each IVR dosing cohort, using paired samples and shift tables. The sample size was too small to perform rigorous statistical comparisons of these transitions.

### Statistical Analyses for Describing Association Between the Structure of the Vaginal Microbiota and TFV PK and PD

We compared the composition and structure of the vaginal microbiota obtained at 1, 2, and 3 months post IVR insertion (V13, 22, and 31, respectively) with the VF TFV concentrations at months 1, 2, and 3 (V11, 20, and 29, respectively). In the main publication of this study, we reported significant differences in TFV VF PK between the continuous and cyclic dosing cohorts only at visits where VF was obtained after a 3-day IVR removal trough in the cyclic group (Thurman et al., 2021). However, for this vaginal microbiota/PK subset analysis, no VF or vaginal tissue samples were obtained immediately after a 3-day IVR removal for the cyclic dosing group. The concentration of TFV in VF was not statistically different between the cyclic and continuous at visits 11, 20, and 29 (Thurman et al., 2021), and therefore, the interrupted and continuous dosing cohorts were combined for the vaginal microbiota and PK analyses in this analysis.

TFV and TFV-DP tissue concentrations were obtained at visit 5 (24, 48, or 72 h post IVR insertion, based on randomization), and we compared these values to the visit 4, pre-IVR insertion vaginal microbiota, as this was the closest sampling available. TFV and TFV-DP tissue concentrations were also measured at the EOT IVR removal visit, V31, and compared to the vaginal microbiota obtained at the same visit (see **Supplementary Table 1**).

The PK data exhibited a log-normal distribution, and therefore log_10_ transformation was applied to not violate the required normality assumption for the statistical comparisons. The difference between the mean log_10_ concentrations of TFV in vaginal tissue and CV aspirate among participants with CST IV vaginal microbiota at months 1, 2, and 3 versus participants with a *Lactobacillus*-dominated vaginal microbiota at months 1, 2, and 3 was compared for CV aspirate using the Bayesian mixed-effect linear model with subject random intercept term analysis for vaginal fluid TFV concentrations and vaginal tissue TFV and TFV-DP concentrations. An association between the concentrations of TFV or TFV-DP (in vaginal tissue or VF) and CST (*Lactobacillus*-dominated CSTs versus CST IVA and CST IVB) was modeled using a Bayesian hierarchical Laplace-binomial model.

For TFV PD in VF, we paired CVL obtained at baseline (V4), month 1 (V11), and month 3 (V29) with vaginal microbiota data obtained at baseline (V4), month 1 (V13), and month 3 (V31). Although we analyzed the change in baseline in HIV inhibition previously using paired methods (Thurman et al., 2021), we used independent group comparisons for differences in HIV inhibition based on vaginal microbiota CST. Since an individual woman’s vaginal microbiota could change CST between baseline and EOT, the CST was based on the CST at the time of sampling. Because these data were not normally distributed, we used the Kruskal–Wallis test to compare differences in HIV inhibition between baseline, month 1, and month 3 for the two vaginal microbiota CSTs (LbD and CST IVA/B). Correlations between TFV PK (TFV concentrations in vaginal fluid) and TFV PD (HIV inhibition by CVL supernatant) were performed using a Spearman correlation coefficient.

We compared untreated (placebo IVR samples) and TFV/LNG IVR baseline, pre-insertion ectocervical and vaginal tissues for p24 antigen production to post-TFV/LNG IVR-treated ectocervical tissues, using independent group comparisons based on the CST at the time of sampling. p24 levels were not normally distributed, and therefore, comparisons were made using the Wilcoxon–Mann–Whitney test for comparison of p24 antigen production between untreated/baseline and posttreatment tissues. We considered a p value or repeated-measure q value of <0.05 significance.

## Results

### Study Population

The first patient was enrolled in October 2017, and the last patient completed the study in December 2018. As previously reported (Thurman et al., 2021), we screened 68 participants and 47 enrolled in the study (37 randomized to the TFV/LNG IVR and 10 randomized to the placebo IVR, used either continuously or cyclically with a 3-day removal after each month). As shown in **Supplementary Figure 1**, all women in the placebo arms completed the study. Seven participants in the TFV/LNG arms did not complete the study. No participant was withdrawn from the study due to adverse events. The demographic data of the randomized participants were previously reported (Thurman et al., 2021) and are summarized in **Supplementary Table 2**. One participant in the cyclic TFV/LNG arm was not included in the enrolled population due to protocol violations.

### Effect of IVR Use on Relative Abundances of Vaginal Microbiota

The composition of the vaginal microbiota was characterized in 167 vaginal swabs from a total of 35 participants. The placebo IVR dosing cohorts (n = 10 participants) each had samples for baseline, month 1, month 2, and month 3 for a total of 40 samples. The TFV/LNG continuous dosing cohort had 18, 16, 14, and 15 samples analyzed at baseline, month 1, month 2, and month 3, respectively, for a total of 63 samples. The TFV/LNG IVR interrupted/cyclic-use cohort had 18, 15, 15, and 16 samples analyzed at baseline, month 1, month 2, and month 3, respectively, for a total of 64 samples. The median total number of 16S rRNA gene sequences was approximately 30,000 per sample. There were 14 TFV/LNG continuous IVR participants and 13 TFV/LNG cyclic IVR participants with adequate paired data at each of the 4 samplings. All 10 placebo IVR participants (continuous and cyclic dosing regimens) had adequate, paired data for all 4 samplings for the safety analyses.

### Effect of IVR Use on the Composition of the Vaginal Microbiota

There were no differences in the composition of the vaginal microbiota (Θ) using bacterial phylotype relative abundances during cyclic or continuous placebo use. The change in the relative abundances and composition of the vaginal microbiota, from baseline, modeled by the Bayesian hierarchical model, did not differ significantly from 0 between baseline pre-insertion and month 1 (all bacterial phylotype p values > 0.16) (**Supplementary Figure 2A**), month 2 (all p values > 0.11) (**Supplementary Figure 2B**), and month 3 (all p values > 0.15) (**Supplementary Figure 2C**) among continuous and interrupted dosing placebo IVR users.

Among women randomized to the TFV/LNG IVR (continuous and cyclic dosing cohorts combined), we found that over 33 bacterial phylotypes were detected in at least 5 women at all samplings. We found significant increases in *Finegoldia* spp. and *Peptoniphilus* spp. (q values < 0.05) and significant decreases in *Prevotella amnii* and *Porphyromonas* spp. (q values < 0.05) between month 1 and baseline (**Supplementary Figure 2D**). By month 2, there were no significant changes from baseline in the relative abundances of any phylotype measured (all q values > 0.11) (**Supplementary Figure 2E**). Finally, at 3 months, we found a significant increase in *Coriobacteriaceae* and significant decreases in *Anaerococcus prevotii* and *Peptoniphilus gorbachii* (q values < 0.05) among TFV/LNG IVR users compared to pre-insertion baseline (**Supplementary Figure 2F**).

When changes in relative abundances of phylotypes between baseline and each month were calculated for the TFV/LNG IVRs continuous dosing group, among the over 19 species that were detected in at least 5 women, there was only one significant change, an increase in *Finegoldia* spp. (q = < 0.001), from baseline to month 1. There were no significant changes in the composition of the microbiota from baseline to month 2 or 3 among continuous TFV/LNG IVR users (all q values > 0.07). Similarly, for the TFV/LNG IVR cyclic cohort, we detected only one species that had significantly increased relative abundance from baseline to the end of treatment at month 3, *Coriobacteriaceae* (q value < 0.001).

### Effect of IVR Use on Structure of the Vaginal Microbiota (CSTs)

We first examined all 7 CSTs (I, II, III, IVA, IVB, IVC, V) defined by VALENCIA (France et al., 2020) and found no significant paired transitions between participants’ CST at baseline and the paired CST at month 1, 2, or 3 for the placebo IVR dosing cohorts (all McNemar p values > 0.60). Similarly, for TFV/LNG IVR users in both dosing cohorts, there were no significant paired changes in the CST between baseline and months 1, 2, and 3 (all p values > 0.11). When the TFV/LNG IVR dosing cohorts (cyclic versus continuous) were analyzed separately, there remained no statistically significant paired transitions between participants’ CST categorization from baseline to each month sampled (all p values > 0.11).

Previous studies examining the impact of topical TFV on the vaginal microbiota (Hillier et al., 2017; Klatt et al., 2017; Thurman et al., 2019) grouped the 7 VALENCIA-defined CSTs into LbD microbiota (CST I, II, III, and V) versus anaerobic, diverse microbiota (CST IVA and IVB). This increases the generalizability of the data as the clinical diagnosis of BV *via* Amsel’s criteria (Amsel et al., 1983) or gram stain/Nugent score (Nugent et al., 1991), widely used in practice, differentiates the vaginal microbiota in these two groups. **Table 1** demonstrates the proportion of women with LbD microbiota versus CST IVA/B microbiota, by treatment group and visit. Of note, 50% of TFV/LNG IVR users and 60% of placebo IVR users had CST IVA/B microbiota at pre-insertion baseline (**Table 1**). We found no significant changes from baseline to month 1, 2, or 3, in the frequency of women with LbD versus CST IVA/B microbiota for any IVR (placebo versus TFV/LNG) and for any dosing regimen (cyclic versus continuous) (all exact McNemar’s p values > 0.60) or when we combined the placebo IVR dosing cohorts (all exact McNemar’s p values > 0.75) or TFV/LNG IVR dosing cohorts (all exact McNemar’s p values > 0.86).

**Table 1:** Frequency table of community state type (CST) groupings at each visit for placebo and tenofovir/levonorgestrel intravaginal ring users (cyclic and continuous dosing cohorts combined).

Month (Visit)	Lactobacillus dominated microbiota (CST I, II, III, V)	Anaerobic, diverse microbiota (CST IVA and IVB)	p value baseline versus each month
**TFV/LNG IVR (cyclic and continuous dosing regimens combined)**			
Baseline (Visit 4)	18	18	REF
Month 1 (Visit 13)	17	14	1.0
Month 2 (Visit 22)	16	13	0.86
Month 3 (Visit 31)	20	11	0.87
**Placebo IVR (cyclic and continuous dosing regimens combined)**			
Baseline (Visit 4)	4	6	REF
Month 1 (Visit 13)	5	5	1.0
Month 2 (Visit 22)	4	6	0.75
Month 3 (Visit 31)	5	5	1.0

### Effect of CST on Residual TFV Concentrations in IVRs at End of Treatment

All returned TFV/LNG IVRs had residual TFV present. Residual amounts of TFV (mean ± standard deviation [SD]) were similar in returned IVRs from participants assigned to TFV/LNG continuous (295 ± 322 mg) and cyclic (362 ± 476 mg) dosing regimens (p = 0.87). In a *post hoc* analysis, we set a threshold of ≥98% drug release (of the 1.15-g loaded TFV in the active IVR) as high *in vivo* release TFV category. Among the 18 continuous TFV/LNG IVR users, 8/18 (44.4%) had high *in vivo* release of TFV from the IVR. Among the 19 cyclic TFV/LNG IVR users, 9/19 (47.4%) had expelled 98% or more of the TFV from the IVR (p = 0.86) by EOT. Combining the two TFV/LNG IVR dosing cohorts, we found no significant differences in age, BMI, partner status, and reported contraceptive use or non-use among women who had high *in vivo* release of TFV versus those who had expected *in vivo* release rates (**Table 2**). There was a higher proportion of women who reported their ethnicity as Hispanic (p = 0.02) and their race as mixed (p = 0.04) or who were recruited at the Dominican Republic research site (p = 0.046) who had high *in vivo* release rates of TFV from the TFV/LNG IVR (**Table 2**).

**Table 2:** Demographic differences between participants based on high versus expected *in vivo* TFV release rates.

Variable	Expected *in vivo* TFV release	High *in vivo* TFV release	p value
	N (%)	N (%)	
**Race**			0.02
American Indian	0	1 (5.9)	
Asian	0	1 (5.9)	
Black	5 (27.8)	2 (11.8)	
Mixed	6 (33.3)	12 (70.9)	
White	7 (38.9)	1 (38.9)	
**Ethnicity**			0.04
Hispanic	7 (38.9)	13 (76.5)	
Non-Hispanic	11 (61.1)	4 (23.5)	
**Research site**			0.05
Dominican Republic	6 (31.6)	12 (70.6)	
Eastern Virginia Medical School	13 (68.4)	5 (29.4)	
**Partner**			0.76
Living with study partner	12 (66.7)	13 (76.5)	
No study partner	3 (16.7)	1 (5.9)	
Not living with study partner	3 (16.7)	3 (17.7)	
**Contraceptive status**			0.22
Abstinence	3 (16.7)	0 (0%)	
Condoms	3 (16.7)	2 (11.8)	
Sterilization of either partner	15 (66.7)	12 (88.2)	
**Microbiota community state type**			
CST IVA/B at Baseline	7 (36.8)	11 (64.7)	0.10
CST IVA/B at Month 1	3 (20.0)	14 (87.5)	<0.01
CST IVA/B at Month 2	0 (0)	16 (94.1)	<0.01
CST IVA/B at Month 3	3 (23.1)	16 (94.1)	<0.01
**Continuous demographic variables**			
Age (mean, standard deviation)	39.4 (5.9)	36.7 (6.2)	0.12
BMI (mean, standard deviation)	26.2 (2.3)	25.9 (3.4)	0.78

Because race and ethnicity are linked to composition and structure of the vaginal microbiota (Ravel et al., 2012), we also compared the CST status at each month and found that while CST at baseline, pre-insertion, was not related to *in vivo* TFV release from the IVR, the development of CST IVA/B during all months of treatment was a significant predictor of high *in vivo* TFV release rate from the IVR, at each month (**Table 2**).

### Effect of the Vaginal Microbiota on Mucosal TFV PK

#### Association Between the Structure of the Vaginal Microbiota and Vaginal Fluid TFV Concentrations

At baseline, we collected samples for vaginal microbiota from all enrolled participants, randomized to TFV/LNG IVR (n = 36), and compared the vaginal microbiota CST at baseline (all levels, CSTs I, II, III, IVA, IVB, V) on all participants with adequate bacterial loads to TFV VF concentrations at 2 and 8 h post insertion, taken the same day. We found no difference in the log_10_ TFV concentrations (ng/mg) immediately after IVR insertion, based on baseline CST (all p values > 0.42). As mentioned above, we added the month 1 and 2 microbiota collections after a protocol amendment. At month 1, there were 31 participants who had paired adequate microbiota and PK sampling. We found no differences in the vaginal fluid TFV concentrations between any of the individual CSTs, except for a significant increase in the TFV VF concentrations at month 1 in women with CST IVB vaginal microbiota, compared to those with CST III vaginal microbiota (p value < 0.04). There were 30 participants with paired microbiota and TFV VF data at month 2 and month 3. Again, analyzed individually, we found no significant differences in TFV VF concentrations among CSTs at months 2 and 3 (all p values > 0.05).

To be comparable with previous TFV PK and vaginal microbiota publications (Hillier et al., 2017; Klatt et al., 2017), we collapsed the 7 individual vaginal microbiota CSTs into LbD CSTs (CSTs I, II, III, V) versus CST IVA/B. When the CSTs were combined in this manner, we found that women with vaginal microbiota comprising diverse anaerobes (CST IVA/B) had significantly higher concentrations of TFV in VF at both months 1 and 2 (**Table 3**) (p values < 0.02), while by the EOT, women with LbD microbiota had higher TFV VF concentrations (p = 0.02).

**Table 3:** Vaginal fluid TFV concentrations (ng/mg) and vaginal tissue TFV (ng/mg) and TFV-DP (fmol/mg) concentrations among TFV/LNG IVR users (cyclic and continuous cohorts combined) with either Lactobacillus dominated (LbD) or CST IVA/B microbiota.

Visit	LbD microbiota				CST IVA/B microbiota				p value
	N	Mean	SD	Median	N	Mean	SD	Median	
TFV in vaginal fluid (ng/mg)									
Month 1	15	1,577	823	1,502	16	3,981	3,337	3,135	0.02
Month 2	14	1,524	1,287	1,364	16	5,926	6,345	3,800	<0.01
Month 3	12	1,647	607	1,423	18	1,463	2,696	351	0.02
TFV in vaginal tissue (ng/mg)									
24 h post IVR insertion	5	40.99	52.39	25.6	7	21.22	18.11	13.72	0.87
48 h post IVR insertion	7	52.9	55.31	25.81	4	19.53	16.41	18.13	0.64
72 h post IVR insertion	6	184.51	191.99	130.3	7	42.88	48.83	23.32	0.87
Month 3	11	189	217	103	19	60	144	5	< 0.01
TFV-DP in vaginal tissue (fmol/mg)									
24 h post IVR insertion	5	408.42	393.73	436.83	7	265.78	277.7	192.57	0.63
48 h post IVR insertion	7	1,936.04	2,621.49	775.32	4	167.05	102.41	151.76	<0.05
72 h post IVR insertion	6	5,566.12	6,319.33	2,833.98	7	4,124.73	6,846.32	2,039.38	0.63
Month 3	11	15,491.9	21,133.3	5,877.7	19	1,457.67	2,599.59	212.38	<0.01

**Figure 1:**
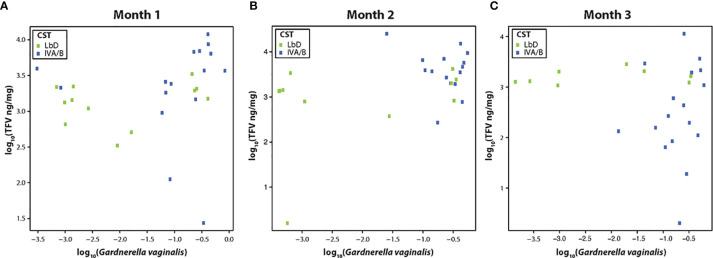
**(A–C)** Correlation of tenofovir (TFV) vaginal fluid concentration with the relative abundance of *Gardnerella vaginalis* at months 1, 2, and 3. Green dots represent participants who had Lactobacillus-dominated (LbD) vaginal microbiota at the time of sampling and their individual TFV vaginal fluid concentration and relative abundance of *Gardnerella vaginalis*. Blue dots represent participants who had diverse anaerobe-dominated (CST IVA/B) vaginal microbiota at the time of sampling and their individual TFV vaginal fluid concentration and relative abundance of *Gardnerella vaginalis*.

We examined the correlation between the relative abundance of *Gardnerella vaginalis* and the TFV VF concentrations at each paired samples (months 1, 2, and 3), due to previous data on the correlation of this species and TFV VF PK (Klatt et al., 2017). There was a significant Spearman correlation between TFV VF concentrations and relative abundance of *G. vaginalis* at month 1 (rho = 0.40, p = 0.03) (**Figure 1A**), a marginally significant correlation between the relative abundance of *G. vaginalis* and TFV VF concentrations at month 2 (rho = 0.38, p = 0.056) (**Figure 1B**), and no significant correlation by month 3 (rho = 0.1, p = 0.96) (**Figure 1C**).

**Figure 2:**
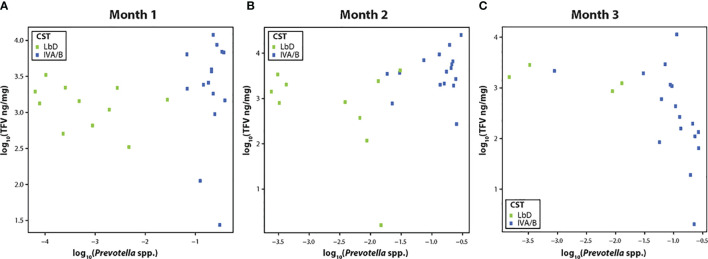
**(A–C)** Correlation of tenofovir (TFV) vaginal fluid concentration with the relative abundance of *Prevotella* species at months 1, 2, and 3. Green dots represent participants who had Lactobacillus-dominated (LbD) vaginal microbiota at the time of sampling and their individual TFV vaginal fluid concentration and relative abundance of *Prevotella* species. Blue dots represent participants who had diverse anaerobe dominated (CST IVA/B) vaginal microbiota at the time of sampling and their individual TFV vaginal fluid concentration and relative abundance of *Prevotella* species.

TFV VF concentrations were not significantly correlated with the relative abundance of *Prevotella* species (spp.) at month 1 (R = 0.30, p = 0.10) (**Figure 2A**), but showed a significant positive correlation at month 2 (R = 0.46, p = 0.02) (**Figure 2B**), and a strong negative correlation by month 3 [(R = -0.70, p < 0.01) (**Figure 2C**)].

#### Association Between the Structure of the Vaginal Microbiota and Vaginal Tissue TFV and TFV-DP Concentrations

There were no significant differences in TFV tissue concentrations among the 6 separate CST between 24 and 72 h post IVR insertion (all p values > 0.15). During the first 72 h post insertion, we measured significantly higher TFV-DP concentrations among women with CST I vaginal microbiota (*L. crispatus*-dominated) compared to CST IVB (p value = 0.03); however, the other groups (CSTs I, II, IVA, IVC) all had similar concentrations of TFV-DP immediately post insertion (all p values > 0.09).

At the EOT, participants with CST III, IVA, and IVB had significantly lower TFV tissue concentrations compared to participants with CST I vaginal microbiota (p values < 0.01). We also measured significantly lower concentrations of TFV-DP in tissue among participants with CST IVA and IVB microbiota compared to participants with CST I vaginal microbiota (p values < 0.01) and CST III vaginal microbiota (p value = 0.05) at EOT.

When the CSTs were collapsed into LbD CSTs (I, II, III, V) versus CST IVA/B, there were no significant differences in the median TFV tissue concentrations at 24, 48, or 72 h post IVR insertion (all p values > 0.64) (**Table 3**). Between 24 and 72 h post IVR insertion, there were no significant differences in TFV-DP concentrations among women who had tissue collected at 24 or 72 h post IVR insertion (all p values > 0.63, **Table 3**). At 48 h post IVR insertion, women with CST IVA/B vaginal microbiota (n = 4) had lower TFV-DP tissue concentrations compared to women with LbD CSTs (n = 7) (p = 0.047, **Table 3**).

By the EOT, participants with LbD vaginal microbiota had significantly higher tissue concentrations of both TFV and TFV-DP (p values < 0.01, **Table 3**). There was a significant positive linear correlation between TFV in vaginal fluid at month 3 and TFV in vaginal tissue (slope = 1.2, p value < 0.001) and TFV-DP in vaginal tissue (slope = 1.13, p values < 0.001) at the EOT. Consistent with our TFV IVR residual data above, the strong linear dependence of TFV and TFV-DP in tissue with TFV in VF was primarily driven by CST IVA/B samples.

**Figure 3:**
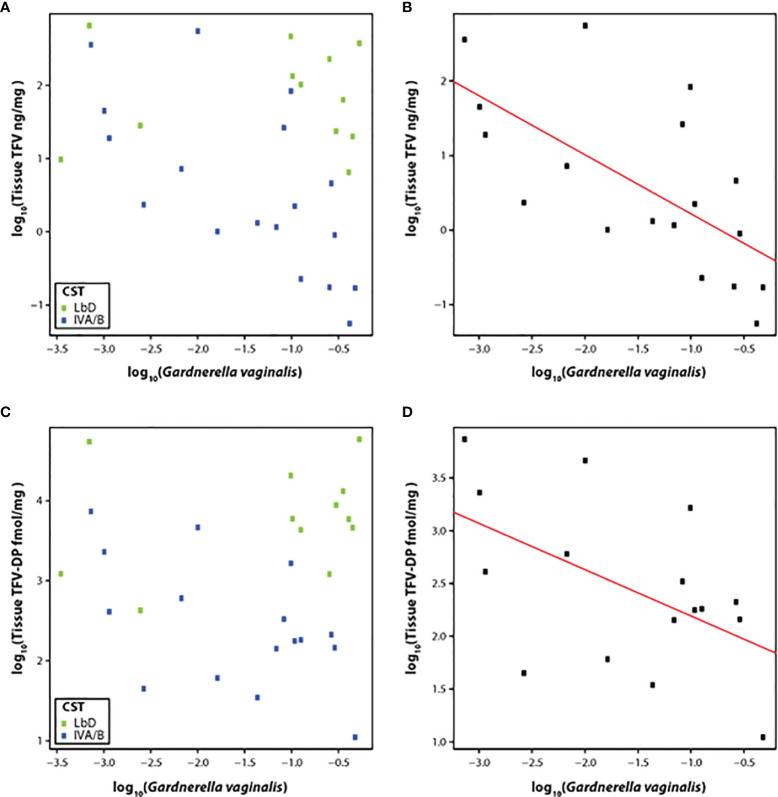
**(A–D)** Correlation of TFV **(A)** and TFV-DP **(C)** tissue concentrations with relative abundance of *G. vaginalis* species and fit of the linear model with CST IV samples at the end of treatment with TFV tissue concentrations **(B)** and TFV-DP tissue concentrations **(D)**. Green dots represent participants who had Lactobacillus-dominated (LbD) vaginal microbiota at the time of sampling and their individual TFV vaginal fluid concentration and relative abundance of *Gardnerella vaginalis*. Blue dots represent participants who had diverse anaerobe-dominated (CST IVA/B) vaginal microbiota at the time of sampling and their individual TFV vaginal fluid concentration and relative abundance of *Gardnerella vaginalis*. Black dots in the linear fit model represent participants who had diverse anaerobe-dominated (CST IVA/B) vaginal microbiota at the time of sampling and their individual TFV vaginal fluid concentration and relative abundance of *Gardnerella vaginalis*.

Therefore, we correlated the relative abundance of *G. vaginalis* with the concentration of TFV in vaginal tissue at the EOT among participants with CST IVA/B vaginal microbiota (n = 19). The slope of the linear model for relative abundance of *G. vaginalis* versus TFV tissue concentration was -0.80 (p < 0.01) (**Figures 3A, B**), although the residual standard error was 0.90. The slope of the linear model for the relative abundance of *G. vaginalis* and the active metabolite, TFV-DP in tissue, was -0.44 (p = 0.04) (residual standard error 0.69) (**Figures 3C, D**).

As we did for TFV VF concentrations, we also fit a model for the relative abundance of *Prevotella* spp. with TFV and TFV-DP tissue concentrations. These linear models were not significant with p values of 0.35 and 0.60 for TFV and TFV-DP, respectively.

### Association Between Vaginal CSTs and TFV PD Modeled *In Vitro*

We previously reported significant increases from pre-insertion baseline, in the inhibitory capacity of the CVL supernatant against HIV-1 *in vitro* at month 1 and month 3 among TFV/LNG IVR users (p values < 0.05) and no significant changes from baseline for placebo IVR users (p values >0.05) (Thurman et al., 2021). There was a strong correlation between the concentration of TFV in VF and the inhibition of HIV-1 *in vitro* by the CVL supernatant (R = 0.87, p < 0.01) among all TFV/LNG IVR users, irrespective of the CST (Thurman et al., 2021).

**Figure 4:**
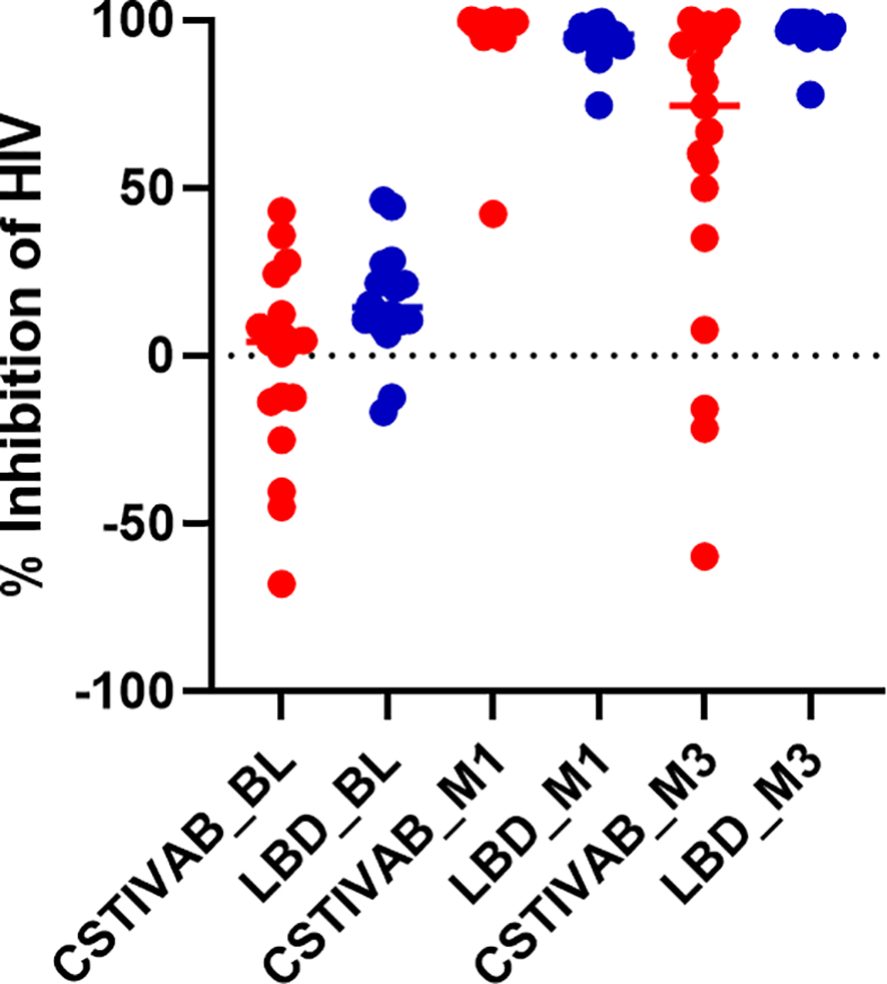
Percent inhibition of HIV *in vitro* by cervicovaginal fluid lavage (CVL) supernatant obtained from study participants using the TFV/LNG IVR at baseline (BL) pre-insertion, month 1 (M1), and end of treatment month 3 (M3). Red dots represent participant data from individuals with diverse anaerobic CST IVA/B microbiota at sampling. Blue dots represent participant data from individuals with Lactobacillus-dominated (LbD) microbiota at sampling. p values < 0.01 paired change from BL in percent HIV inhibition at months 1 and months 3 for both LbD and CST IVA/B groups.

When the PD data were stratified by IVR and CST, we again confirmed no significant change in HIV inhibition among placebo IVR users (p values > 0.48) with either LbD or CST IVA/B vaginal microbiota. For TFV/LNG IVR users, there was a significant increase from baseline at months 1 and 3 in the median inhibitory capacity of the CVL against HIV-1 *in vitro*, whether participants had CST IVA/B or LbD vaginal microbiota at the sampling visit (all p values < 0.01) (**Table 4**, **Figure 4**). At month 1, median HIV-1 inhibition was significantly higher among women with CST IVA/B vaginal microbiota (98.75%) compared to those with LbD CST (95.61%) (p = 0.03). By month 3, while all women maintained significant increases in HIV-1 inhibition compared to baseline, those with CST IVA/B vaginal microbiota had significantly lower median HIV-1 inhibition by CV fluids (74.42%) compared to women with LbD vaginal microbiota (97.18%) (p < 0.01) (**Table 4**). The PK/PD correlation between TFV concentration in VF and HIV inhibition *in vitro* by VF supernatant remained significant when the data were categorized by CST IVA/B (R = 0.89, p < 0.001) or LbD CSTs (R = 0.81, p < 0.01).

**Table 4:** Change from baseline in HIV inhibition by vaginal fluid based on IVR and community state type (CST).

IVR and CST group	Pre-insertion baseline				Month 1				Month 3				p value
	N	Mean	SD	Median	N	Mean	SD	Median	N	Mean	SD	Median	
TFVLNG IVR (continuous and cyclic cohorts combined)													
CST IVA/B	18	-2.46	28.85	4.21	17	94.87	13.69	98.75	19	57.71	47.63	74.42	< 0.01
LbD	18	16.14	15.85	14.38	13	93.88	6.63	95.61	11	95.68	6.15	97.18	< 0.01
Placebo IVR (continuous and cyclic cohorts combined)													
CST IVA/B	4	-7.57	29.96	-15.27	5	-3.04	54.69	32.34	5	-28.79	48.03	-20.68	0.48
LbD	6	2.45	11.5	5.9	5	-26.54	82.45	-6.94	5	-0.85	31.91	4.99	0.81

**Figure 5:**
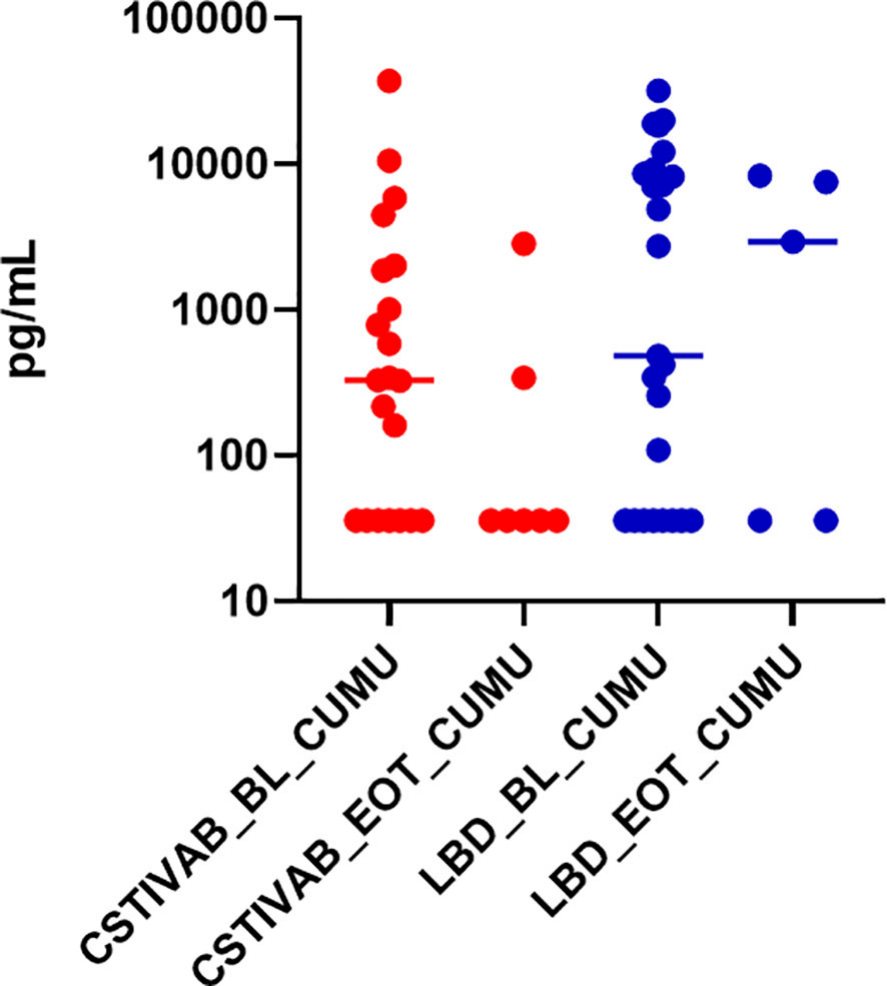
Cumulative (CUMU) p24 antigen production from cervicovaginal (CV) tissue biopsies obtained from all participants at baseline and from participants using the placebo IVR (BL). CUMU p24 antigen production from CV tissue biopsies obtained from participants using the TFV/LNG IVR at end of treatment (EOT). Red dots represent participant data from individuals with diverse anaerobic CST IVA/B microbiota at sampling. Blue dots represent participant data from individuals with Lactobacillus-dominated (LbD) microbiota at sampling. p values > 0.05 for change in p24 antigen production from tissues at EOT compared to BL.

For the PD tissue modeling studies, we previously reported that after the TFV/LNG treatment, there was a 10–20-fold reduction in median p24 antigen production from biopsied ectocervical tissues challenged with HIV_BaL_ *ex vivo*, compared to untreated (placebo IVR) and baseline (visit 3) tissue samples (Thurman et al., 2021). When we stratify the p24 data by CST at the time of tissue sampling, we end up with a small sample size in each subcategory, and although we see a reduction of p24 from baseline with both CST IVA/B and LbD vaginal microbiota, the differences were not statistically significant (**Figure 5**; p values >0.09 for CST IV and >0.55 for LbD).

## Discussion

The CONRAD ENRICH Study (protocol A15-138) was a placebo-controlled, follow-up study of the TFV/LNG IVR, used either cyclically or continuously for up to 90 days. In our first study (CONRAD A13-128), women used placebo, TFV, or TFV/LNG IVRs for approximately 2 weeks, inserting the ring immediately after menses and removing it prior to the next menses (Thurman et al., 2019). In this first-in-human study, the rings were found to be safe and met prespecified PK/PD benchmarks (Thurman et al., 2019). In terms of safety, the findings of the current extended-use study (CONRAD 138) are consistent with our previous report (Thurman et al., 2019). Use of the placebo or TFV/LNG IVRs had few to no clinically significant impacts on the composition and structure of the vaginal microbiota. While there were a few significant increases or decreases in individual bacterial phylotypes at various months, there were no consistent, significant changes in CSTs from baseline each month and the effect of TFV/LNG IVR on the composition of the vaginal microbiota was not different from that of placebo IVR. Most women using the TFV/LNG IVR also reported a decrease in menses or no change in their bleeding patterns (Thurman et al., 2021). This finding was encouraging since breakthrough menstrual bleeding, often seen with high-dose progestin-only contraceptives (Teunissen et al., 2014; Pillay et al., 2017; Sznajder et al., 2017; Cohen et al., 2019), may affect the composition of the vaginal microbiota (Ravel et al., 2012).

There was no evidence that IVR use increased the incidence of BV and no signals that IVR use caused any detrimental impact on the composition and structure of the vaginal microbiota when women used the IVR for the full 90-day duration. These findings are in agreement with those of a recent study of the TFV-based rings in Kenyan women (CONRAD 144 subset study) (Dabee et al., 2020). These reassuring vaginal microbiota safety data are in accordance with previous data, albeit using less sensitive culture and gram-stain methods, showing no significant changes in vaginal bacteria among a cohort of healthy, sexually active women using various contraceptive IVRs (Roy et al., 1981; Davies et al., 1992; Roumen et al., 1996; De Seta et al., 2012; Huang et al., 2015) for up to 12 months. Recently, others examined the vaginal microbiota among ethinyl estradiol (EE) and etonogestrel (ETG) contraceptive IVR users longitudinally, using either fluorescent *in situ* hybridization (FISH) or 16S RNA gene amplicon sequencing (Crucitti et al., 2018; Kestelyn et al., 2018). Among this cohort, with a 48% baseline prevalence of BV in Rwanda, the vaginal Nugent score (Nugent et al., 1991) decreased after using the contraceptive IVR (Kestelyn et al., 2018) and vaginal concentrations of *Lactobacillus* species increased and *G. vaginalis* and *A. vaginae* decreased significantly from baseline over the 12-week observation period (Crucitti et al., 2018). Two studies support that EE plus ETG delivered vaginally as the contraceptive IVR among young women has a more beneficial effect on the vaginal microbiota compared to EE plus progestin delivered systemically as an oral contraceptive pill (Veres et al., 2004; De Seta et al., 2012). Our data represent the first study using molecular characterization of the composition of the vaginal microbiota using 16S rRNA gene amplicon sequencing to describe the effect of a progestin-only MPT IVR on the vaginal microbiota. Although the sample size was small, these findings support that topical micro-dose LNG does not adversely affect the composition and structure of the vaginal microbiota.

In our first study, we found that participants using the TFV or TFV/LNG IVRs for approximately 2 weeks with either LbD or CST IV vaginal microbiota experienced high concentrations of TFV in VF and TFV and TFV-DP in vaginal tissue (Thurman et al., 2019), exceeding benchmarks previously established using peri-coitally dosed TFV vaginal gel for HIV-1 prevention (Parikh et al., 2009; Dobard et al., 2011; Karim et al., 2011; Kashuba et al., 2015) and HSV-2 prevention (Abdool Karim et al., 2015). These new data are in accordance with our initial findings but give new insight into how this MPT ring functions when women use it for the full 90-day duration. The TFV/LNG IVR provided very high CV mucosal concentrations of TFV to all users. Although there were significant differences in TFV VF concentrations between participants with LbD and CST IVA/B microbiota at each month of use, mean and median VF TFV concentrations exceeded 1 ng/mg (approximately 1,000 ng/ml) at all visits.

In our first study of the TFV MPT IVRs (Thurman et al., 2019), we measured TFV concentrations in the CV aspirate in ng/mL, as was done in the CAPRISA 004 TFV vaginal gel study (Karim et al., 2011; Kashuba et al., 2015). Since then, our PK central laboratory and others found that the CV concentrations of TFV are more accurately defined by measuring the exact weight of secretions obtained from vaginal swabs and reporting concentrations of fluid as ng/mg rather than ng/mL of CV aspirate. We used the same PK central lab for both our initial (Thurman et al., 2019) and current studies (Thurman et al., 2021). While there are no established benchmarks for HIV-1 infection prevention for the TFV/LNG IVR in women, it is worth noting that a CV aspirate concentration of 100 ng/ml conferred an estimated 65% protection, while a CV aspirate of over 1,000 ng/ml reduced the risk of HIV-1 acquisition by 76% when women used peri-coitally dosed TFV vaginal gel in the CAPRISA 004 study (Karim et al., 2011; Kashuba et al., 2015).

Our PD modeling data also support that the TFV VF concentrations achieved provided significant increases in median HIV-1 inhibition, for women with either LbD CSTs (96% and 97%, at months 1 and 3, respectively) or CST IVA/B (99% and 74% at months 1 and 3, respectively) vaginal microbiota over the median baseline (14% and 4% for LbD and CSTIV, respectively), pre-insertion HIV-1 inhibition levels. While CST IVA/B had higher HIV-1 inhibitory activity at month 1 and lower HIV-1 inhibitory activity at month 3 compared to LbD CSTs, it is not known whether these differences modeled *in vitro* are clinically significant, since both are significantly higher than baseline activity.

The correlations between TFV VF PK and CST IVA/B-associated species (*G. vaginalis* and *Prevotella* spp.) support interactions between the composition of the vaginal microbiota, *in vivo* TFV release from the IVR, and CV TFV PK. During the first 2 months of use, individuals had a significant positive correlation between BV-associated bacteria (CST IVA/B) and increased *in vivo* release of TFV from the IVR, resulting in high TFV VF concentrations. By month 3, individuals with high concentrations of *Prevotella* spp. had a significantly inverse relationship with TFV VF concentrations. Similarly, individuals with high levels of *G. vaginalis* showed lower TFV and TFV-DP concentrations in tissue at the EOT.

Although all users had residual TFV present in the IVRs at the EOT, the lower concentrations of TFV in VF and tissue may be due to the initial higher-than-expected *in vivo* release of TFV from the IVR in women with CST IVA/B vaginal microbiota with possible depletion of TFV from the IVR by the EOT. Alternatively, it is also possible that the previously demonstrated competing metabolism of TFV by BV-associated bacteria (e.g., *G. vaginalis* and *Prevotella* spp.) (Taneva et al., 2018) begins to impact mucosal concentrations at the end of TFV/LNG IVR use. Our data on the negative correlation of *Prevotella* spp. and TFV VF concentrations at EOT are consistent with previous *in vitro* experiments showing that *Prevotella* spp. and *G. vaginalis* metabolize TFV and compete for its uptake by Jurkat cells in culture (Klatt et al., 2017). However, the positive correlations between TFV concentrations in VF and *G. vaginalis* and *Prevotella* spp. levels at months 1 and 2 do not support the hypothesis of a direct metabolizing effect by the vaginal microbiota.

Based on preclinical, *in vitro* release data (Johnson et al., 2012), and the well-described association between higher vaginal pH and a vaginal microbiota lacking *Lactobacillus* spp. and comprising strict and facultative anaerobes (Hemalatha et al., 2013; Hoffman et al., 2017), we hypothesize that vaginal pH is a main factor driving *in vivo* TFV release. *In vitro* and animal data in sheep suggest that the solubility of TFV increases with pH (Johnson et al., 2012). When higher pH vaginal fluid penetrates the hydrating IVR, more TFV will dissolve from the formulated paste inside the IVR (Johnson et al., 2012). The higher concentration of solubilized TFV is then free to permeate out of the IVR reservoir, which is already being driven by osmotic pressure forces generated from the osmotic attractant (glycerol) co-formulated in the starting core paste (Johnson et al., 2012). Unfortunately, we only measured vaginal pH at the screening visit in this study and therefore cannot directly confirm this relationship.

Although we correlated high TFV *in vivo* release with participant race, ethnicity, and study site, none of these factors showed a significant overall correlation in multiple logistic regression analyses. This further supports the hypothesis of higher TFV release associated with BV vaginal microbiota (Ravel et al., 2012). Given what we know about the uptake and degradation of TFV by CSTIV vaginal microbiota (Klatt et al., 2017; Taneva et al., 2018), an increased release in the presence of this type of microbiota would seem an ideal response by the TFV IVR, supported by the fact that women with BV-like vaginal microbiota (CST IVA/B) had higher VF concentrations of TFV at the month 1 and 2 samplings. Higher release rates, however, may point to the need for higher TFV ring loading, if the desired duration is 90 days, as shown by the decreased concentrations of TFV after 3 months of use. Of note, in spite of differences in TFV CV levels, our PD modeling data indicated that the HIV inhibitory activity of CV fluid is significantly augmented throughout the 90 days of IVR use in all women regardless of CST.

For the vaginal tissue PK sampling, due to logistical issues, we only sampled women within 72 h of IVR insertion and then again at the EOT. Within 72 h of IVR insertion, local TFV tissue concentrations were high with non-significant differences according to the type of vaginal microbiota and minimal impact on TFV-DP concentrations with large data dispersion.

At the EOT, as TFV IVR load may have been depleted due to the higher release rate in women with CST IVA/B vaginal microbiota, we observed that women with this type of microbiota had significantly lower concentrations of TFV and TFV-DP in tissue compared to women with LbD CSTs. Women with LbD CSTs had mean and median TFV-DP tissue concentrations exceeding 1,000 fmol/mg at month 3. At the end of treatment, women with CST IVA/B vaginal microbiota had mean TFV-DP tissue concentrations of 1,458 fmol/mg, but the median of 211 fmol/mg is below the threshold established in non-human primates for TFV vaginal gel (Parikh et al., 2009; Dobard et al., 2011). This dispersion suggests the existence of two subcohorts in that group: one with normal levels and one of very low levels likely reflecting depletion of TFV from their rings at EOT.

Not surprisingly, we found that TFV concentrations in VF were strongly and positively correlated with TFV and TFV-DP tissue concentrations. TFV is taken up by the vaginal tissue and metabolized in mucosal cells to the active metabolite, TFV-DP (Cottrell and Kashuba, 2014). By the EOT, TFV VF concentrations were significantly lower in women with CST IVA/B vaginal microbiota. By month 3, among the CST IVA/B cohort, there were significant negative correlations between TFV and TFV-DP tissue concentrations and the relative abundances of *G. vaginalis*, but not *Prevotella* spp. Similar findings were reported for a TFV film formulation studied in FAME-004 (Hillier et al., 2017).

Although appropriately powered for the primary endpoints, the CST subcategorization study yielded small sample sizes for tissue HIV challenge assays, and our PD modeling tissue data are not as strong as those in VF. The PD tissue model requires immediate fresh tissue biopsies, and therefore, we could only sample participants enrolled at our US site for this endpoint, reducing the sample size by half. By categorizing the study population based on two CSTs, we further reduced the sample size. We previously reported a 10–20-fold reduction in HIV p24 antigen tissue production after TFV/LNG IVR treatment among all users (Thurman et al., 2021). The p24 data are often highly variable (Richardson-Harman et al., 2012), and although women with CST IVA/B vaginal microbiota had general decreases in median p24 antigen production and lower infection rates compared to baseline (28% vs. 68% infected tissues, respectively), the differences were not statistically significant. Thus, while our VF PD data support significant increases in HIV inhibitory activity among all users, our tissue PD modeling data, subcategorized by CST, are non-conclusive, and this is a limitation of the study. Unlike the large cohort (n = 688) reported by Klatt et al. (2017), our past (Thurman et al., 2019) and current TFV MPT ring studies and the FAME 04 TFV film subanalysis (Hillier et al., 2017) provide quantification of TFV and TFV-DP concentrations in genital tissue biopsies, which was not feasible in the larger CAPRISA 004 cohort (Klatt et al., 2017).

Although we sampled participants for VF TFV concentrations at several times throughout IVR use, we only have concurrent vaginal microbiota and PK data at monthly intervals for TFV vaginal fluid concentrations and only at two time points for TFV and TFV-DP tissue concentrations. Larger cohorts with more frequent tissue sampling would be needed in future studies to confirm our findings.

Unlike our first-in-women study (Thurman et al., 2019), participants in this trial used the IVR for the full intended duration of use, were allowed to enroll with asymptomatic BV (over 50% of participants had CST IVA/B vaginal microbiota, which is similar to that associated with the diagnostic of BV, at the time of IVR insertion), and were permitted to have vaginal intercourse, except after vaginal biopsies, making these data more generalizable to larger populations. Furthermore, the study used advanced methodology to characterize and quantify the composition and structure of the vaginal microbiota and correlate its changes with TFV and its active metabolite, TFV-DP, levels in fluids and tissues. To our knowledge, this is the first study on the impact of a locally delivered micro-dose of LNG on the vaginal microbiota.

Our data support that the vaginal microenvironment and microbiota impact *in vivo* TFV release from the IVR. CST IVA/IVB and BV-associated bacteria, possibly through concomitantly elevated vaginal pH, increased the release of TFV, resulting in higher CV concentrations as seen in the first 2 months of observation. Given the postulated effect of these bacteria taking up and degrading TFV and consequently decreasing its efficacy (Klatt et al., 2017), an increased *in vivo* TFV release rate from the IVR, although unexpected, represents a likely desirable positive change countering the potential negative effect of BV-associated bacteria. With the current original TFV loading, the IVR provided high levels of TFV and TFV-DP in the mucosal environment associated with LbD vaginal microbiota and normal pH but ran low at EOT in the presence of CST IV and its associated higher pH (Ravel et al., 2012). It is also possible that in the presence of lower TFV concentrations, the reported degradation of TFV by BV-associated bacteria (Klatt et al., 2017) further compounds the problem. Additional loading of TFV would solve this issue enabling a “smart” response of the IVR when applied in the presence of BV or other non-optimal states of the vaginal microbiota.

This placebo-controlled study among healthy sexually active women using the TFV/LNG IVR for 90 days confirms that this IVR had no adverse impact on the composition and structure of the vaginal microbiota, using state-of-the-art methods to characterize it. In general, TFV levels in the CV compartment were high and compatible with HIV inhibition. Notably, TFV levels varied according with the structure of the vaginal microbiota (CST). In women with LbD vaginal microbiota, TFV levels in fluids and tissues were high, as previously reported, and were associated with high HIV inhibitory capacity. In women with CST IV vaginal microbiota, TFV levels were higher in the first 2 months of sampling but decreased and were lower than those in LbD vaginal microbiota at EOT (~3 months). With increased loading of TFV, the IVR would be reacting to BV with increased release without potentially exhausting its payload at the end of the dosing period. This property is unique to this polyurethane-based TFV ring and would solve the problem of decreased efficacy associated with BV reported for TFV gel (Klatt et al., 2017), TFV film (Hillier et al., 2017) and postulated for other microbicides (Taneva et al., 2018).

We propose that TFV/LNG IVRs will fill an important gap as an MPT that women, particularly those in less developed countries, can utilize to protect themselves from HIV-1 and unintended pregnancies.

## Data Availability Statement

The original contributions presented in the study are publicly available in NCBI under accession number PRJNA785597.

## Ethics Statement

The studies involving human participants were reviewed and approved by the Advarra Institutional Review Board (IRB) (#Pro00022358) for the EVMS site and Comisiòn Nacional de Bioetica (#030-2017) for the PROFAMILIA site. The patients/participants provided their written informed consent to participate in this study.

## Author Contributions

AT conducted the clinical trial, managed the participants, interpreted the data, and drafted and reviewed the manuscript. JR and PG analyzed the microbiota samples, analyzed the microbiome and PK data, interpreted the data, and reviewed and contributed to the manuscript. MM analyzed the PK fluid and tissue samples, analyzed the PK data, interpreted the data, and reviewed and contributed to the manuscript. LO, TJ, MP, and MC performed the PD endpoint analyses and sample processing, analyzed the vaginal fluid and vaginal tissue PD data, interpreted the data, and reviewed and contributed to the manuscript. GD conceived the study, is the PI of the funding source, analyzed the microbiome and PD and PK data, interpreted the data, and reviewed and contributed to the manuscript. All authors contributed to the article and approved the submitted version.

## Funding

This study was supported by the United States Agency for International Development (USAID) and (PEPFAR) under Cooperative Agreements (AID-OAA-A-10-00068 and AID-OAA-A-14-00010). JR was supported by NIAID, NIH, under award number U19AI084044. The contents are the sole responsibility of the authors and do not necessarily reflect the views of their institutions, USAID, PEPFAR, or the United States Government. Gilead Sciences donated the tenofovir active pharmaceutical ingredient. The funders had no role in the study design, data collection and analysis, decision to publish, or preparation of the manuscript.

## Conflict of Interest

The authors declare that the research was conducted in the absence of any commercial or financial relationships that could be construed as a potential conflict of interest.

## Publisher’s Note

All claims expressed in this article are solely those of the authors and do not necessarily represent those of their affiliated organizations, or those of the publisher, the editors and the reviewers. Any product that may be evaluated in this article, or claim that may be made by its manufacturer, is not guaranteed or endorsed by the publisher.

## Supplementary Material

Supplementary Table 1
**|** Schedule of evaluations for microbiome, pharmacokinetics and pharmacodynamics endpoints in the CONRAD 138 ENRICH trial. CV, cervicovaginal; IVR, intravaginal ring; PK, pharmacokinetics; TFV, tenofovir; TFV-DP, tenofovir-diphosphate

Supplementary Table 2
**|** Demographic and baseline characteristics, treated population. * = One participant excluded from the analysis population due to protocol violations.
