# Supplementary material for: Vaginal Microbiota and Mucosal Pharmacokinetics of Tenofovir in Healthy Women Using a 90-Day Tenofovir/Levonorgestrel Vaginal Ring
Source: Front Cell Infect Microbiol. 2022 Mar 8;12:799501. doi: 10.3389/fcimb.2022.799501 (PMC8957918; doi:10.3389/fcimb.2022.799501)
Supplement: Supplementary Table 1 — | Schedule of evaluations for microbiome, pharmacokinetics and pharmacodynamics endpoints in the CONRAD 138 ENRICH trial. CV, cervicovaginal; IVR, intravaginal ring; PK, pharmacokinetics; TFV, tenofovir; TFV-DP, tenofovir-diphosphate [file Table_1.docx]

**Supplemental Table 1 |** Schedule of evaluations for microbiome, pharmacokinetics and pharmacodynamics endpoints in the CONRAD 138 ENRICH trial

|  | | **Visit 1**  Screen | **Visit 2** | **Visit 3**  Base-line | **Visit 4**  IVR Insertion | **Visit 5**  24, 48, or 72h post insertion | **Visits**  **8,17,26** | **Visits**  **11,20,29** | **Visits**  **13,22,31** | **Visits**  **14,23** | **Visit 32**  48h, 72h, or 5d post IVR removal |
| --- | --- | --- | --- | --- | --- | --- | --- | --- | --- | --- | --- |
| Approximate Day of Use | |  |  |  | 1 | 2, 3, 4 | 10, 42, 73 | 21, 53, 84 | 28, 59, 90 | 32, 63 |  |
| IVR In Situ or Removed for Cyclic Dosing Cohort | |  |  |  | IVR Insertion | In Situ | In Situ | In Situ | Day of removal | Reinsertion of IVR after 3d removal | Removed |
| Informed consent | | ✓ |  |  |  |  |  |  |  |  |  |
| Microbiome | CV Fluid |  |  |  | ✓ (pre-insert) |  |  |  | ✓ |  |  |
| PK | CV Tissue – Biopsy TFV, TFV-DP |  |  |  |  | ✓ |  |  | ✓  (V31) |  | ✓ |
|  | CV Fluid TFV |  |  |  | ✓  (2,8h) | ✓ | ✓ | ✓ |  | ✓ | ✓ |
| In Vitro PD Modeling | CVL for HIV Inhibition |  |  |  | ✓ (pre-insert) |  |  | ✓ (V11, V29) |  |  |  |
|  | CV Tissue –Biopsy HIV-1 infection (EVMS site only) |  |  |  | ✓ (pre-insert) |  |  |  | ✓  (V31) |  |  |
| IVR Removal (Complete IVR processing at V31) | |  |  |  |  |  |  |  | ✓ |  |  |

CV=cervicovaginal; IVR=intravaginal ring; PK=pharmacokinetics; TFV=tenofovir; TFV‑DP=tenofovir‑diphosphate
