# Supplementary material for: Vaginal Microbiota and Mucosal Pharmacokinetics of Tenofovir in Healthy Women Using a 90-Day Tenofovir/Levonorgestrel Vaginal Ring
Source: Front Cell Infect Microbiol. 2022 Mar 8;12:799501. doi: 10.3389/fcimb.2022.799501 (PMC8957918; doi:10.3389/fcimb.2022.799501)
Supplement: Supplementary Table 2 — | Demographic and baseline characteristics, treated population. * = One participant excluded from the analysis population due to protocol violations. [file Table_2.docx]

**Supplemental Table 2 |** Demographic and baseline characteristics, treated population

|  | **TFV/LNG IVR**  **Continuous and Cyclic Cohorts**  **(n = 36*)** | **Placebo IVR**  **Continuous and Cyclic Cohorts**  **(n = 10)** |
| --- | --- | --- |
| **Age (years)** |  |  |
| Median (Q1, Q3) | 37.5 (34.0, 42.0) | 34.0 (29.0, 36.0) |
| **Body Mass Index (kg/m2)** |  |  |
| Median (Q1, Q3) | 26.5 (25.0, 28.0) | 25.0 (23.0, 28.0) |
| **Ethnicity** |  |  |
| Hispanic/Latina | 21 (58.3%) | 7 (70.0%) |
| Not Hispanic/Latina | 15 (41.7%) | 3 (30.0%) |
| **Race** |  |  |
| American Indian or Alaska Native | 1 (2.8%) | 0 |
| Asian | 1 (2.8%) | 0 |
| Black/African American | 7 (19.4%) | 3 (30.0%) |
| White | 8 (22.2%) | 2 (20.0%) |
| Mixed | 19 (52.8%) | 5 (50.0%) |
| **Education (years)** |  |  |
| Median (Q1, Q3) | 12.0 (8.5, 14.5) | 11.5 (10.0, 15.0) |
| **Contraceptive Method Used in Study** |  |  |
| Sterilization of either partner | 28 (77.8%) | 7 (70.0) |
| Abstinence | 3 (8.3%) | 1 (10.0%) |
| Non-spermicidal condoms | 5 (13.9%) | 2 (20.0%) |
| **Study Partner Status** |  |  |
| Living with study partner | 26 (72.2%) | 6 (60.0%) |
| Not living study with partner | 6 (16.7%) | 0 |
| No study partner | 4 (11.1%) | 4 (40.0%) |
| **Was the participant ever pregnant?** |  |  |
| Yes | 34 (94.4%) | 10 (100%) |
| No | 2 (5.6%) | 0 |

* = One participant excluded from the analysis population due to protocol violations.
