# Supplementary figures and images for: Vaginal Microbiota and Mucosal Pharmacokinetics of Tenofovir in Healthy Women Using a 90-Day Tenofovir/Levonorgestrel Vaginal Ring
Source: Front Cell Infect Microbiol. 2022 Mar 8;12:799501. doi: 10.3389/fcimb.2022.799501 (PMC8957918; doi:10.3389/fcimb.2022.799501)

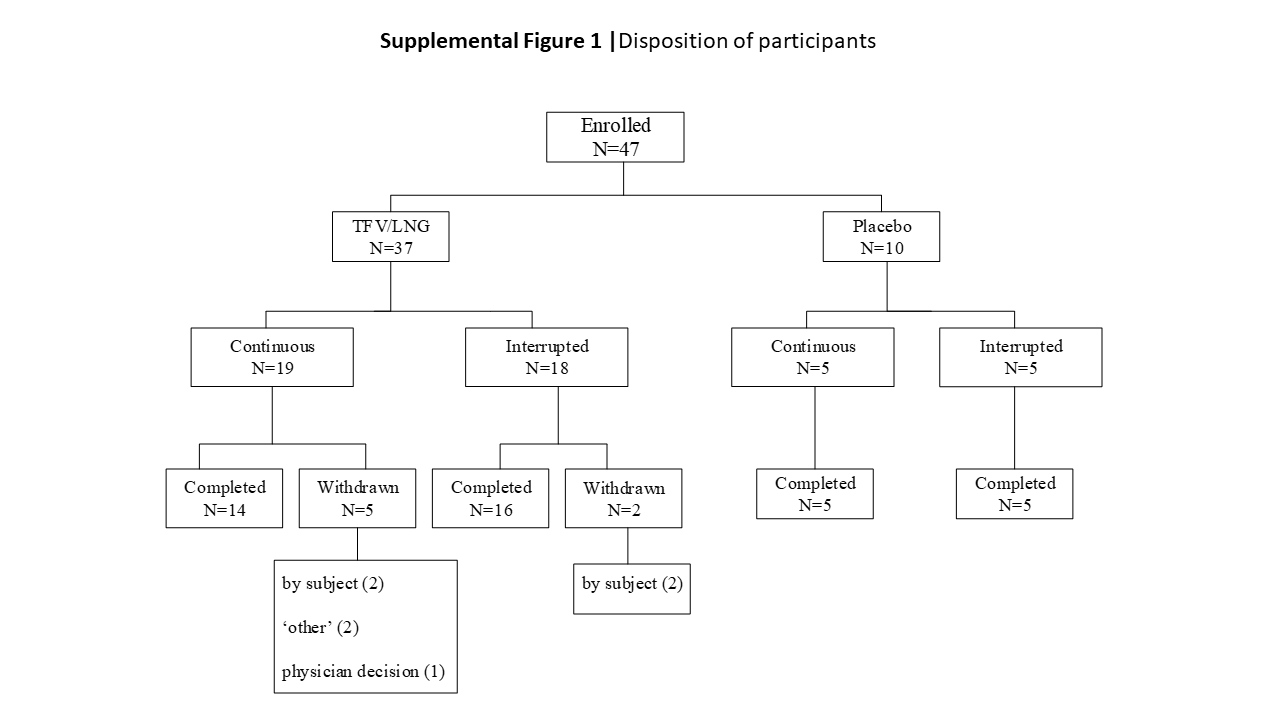

Supplement: Supplementary file 3 [file Image_1.tif]

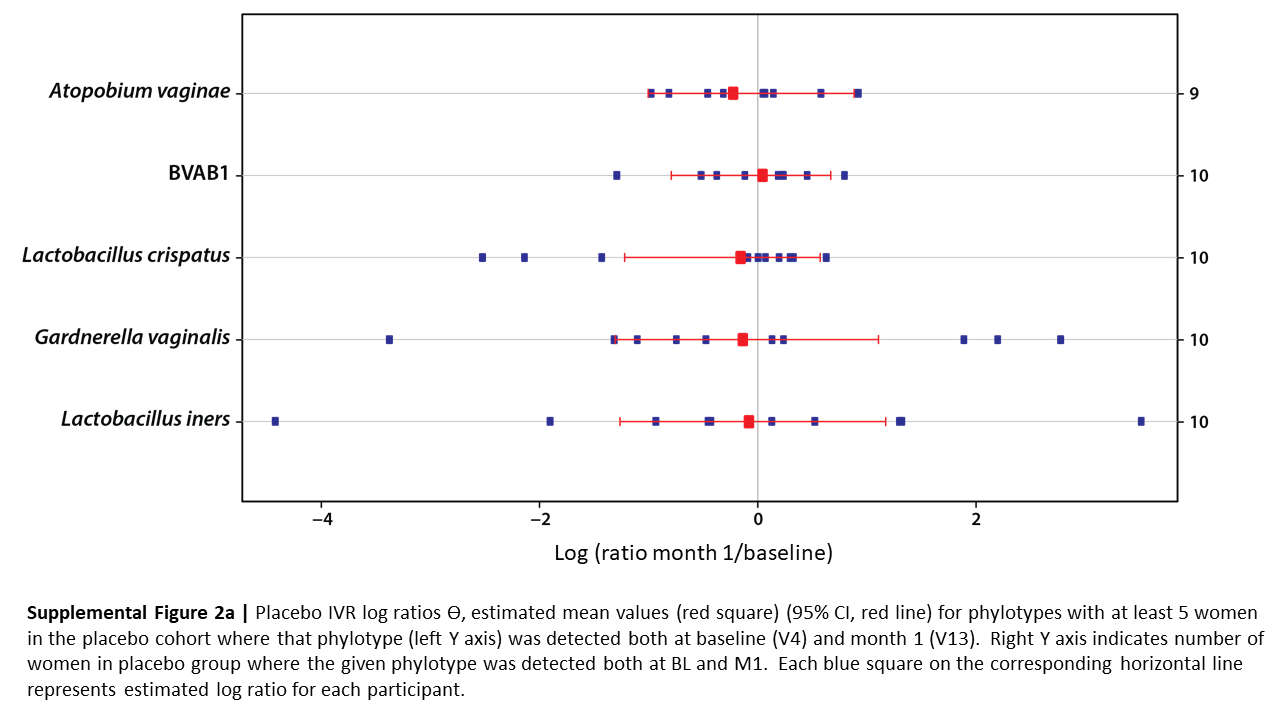

Supplement: Supplementary file 4 [file Image_2.tif]

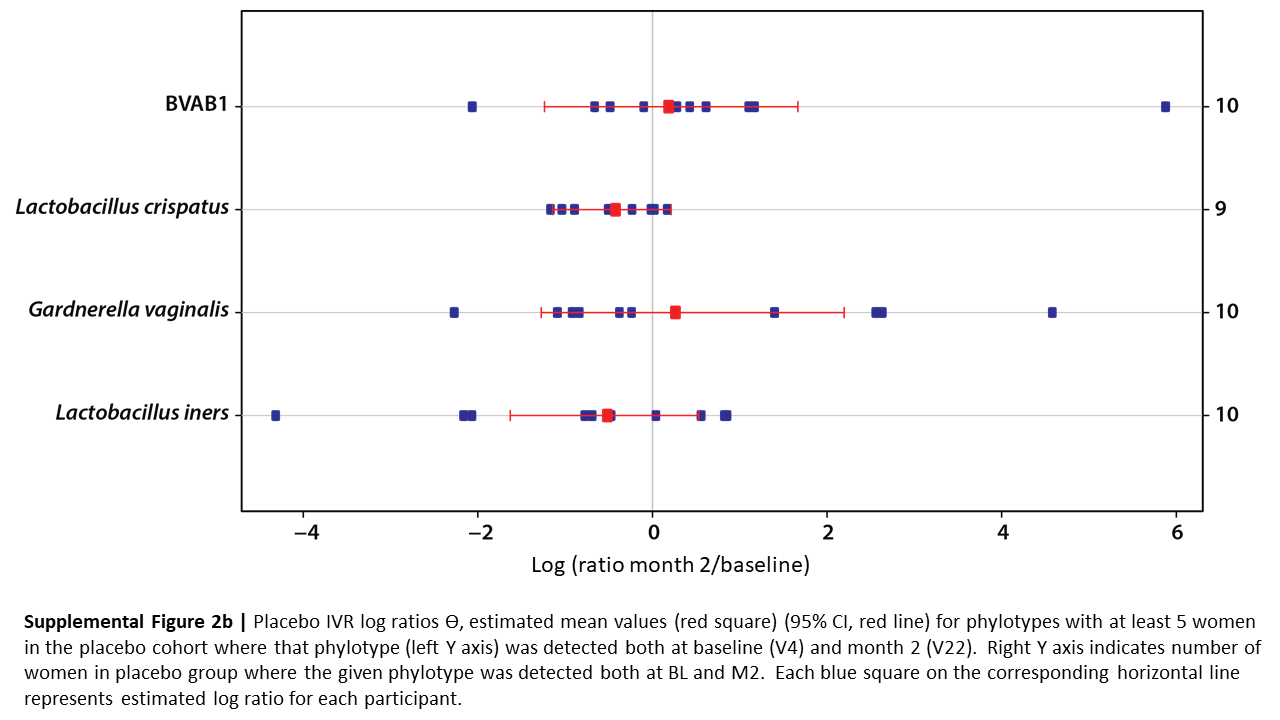

Supplement: Supplementary file 5 [file Image_3.tif]

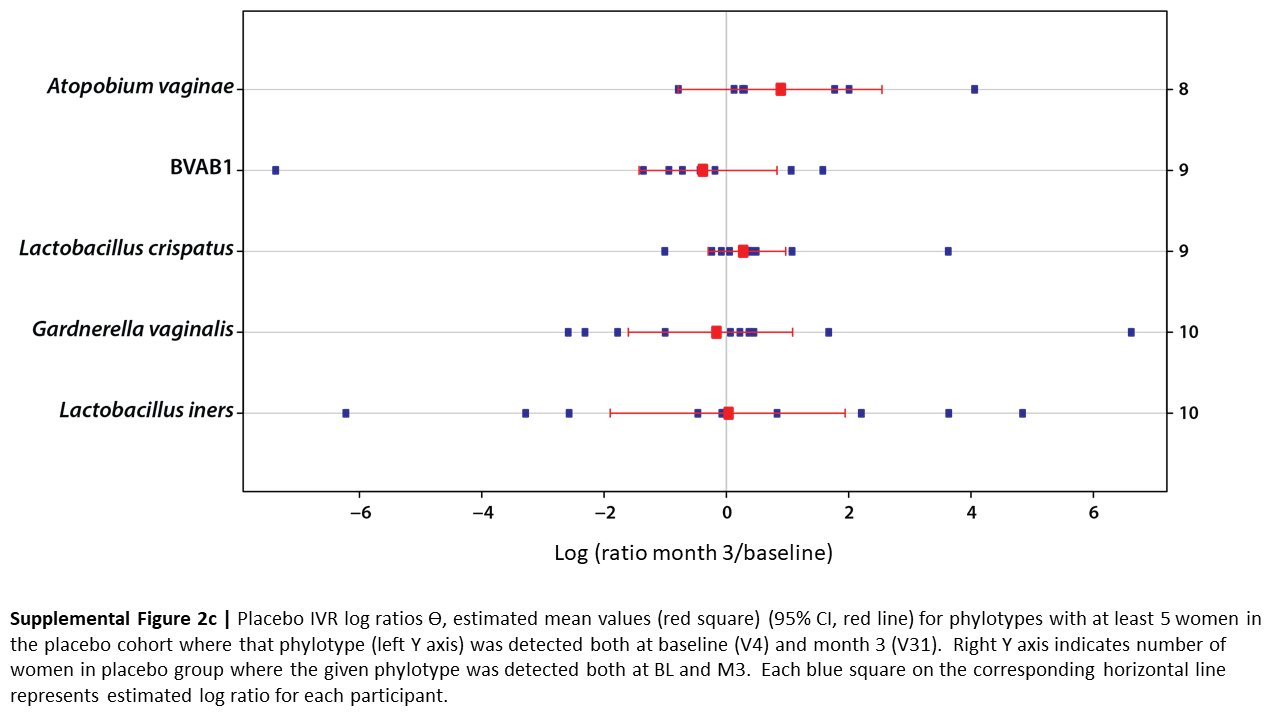

Supplement: Supplementary file 6 [file Image_4.tif]

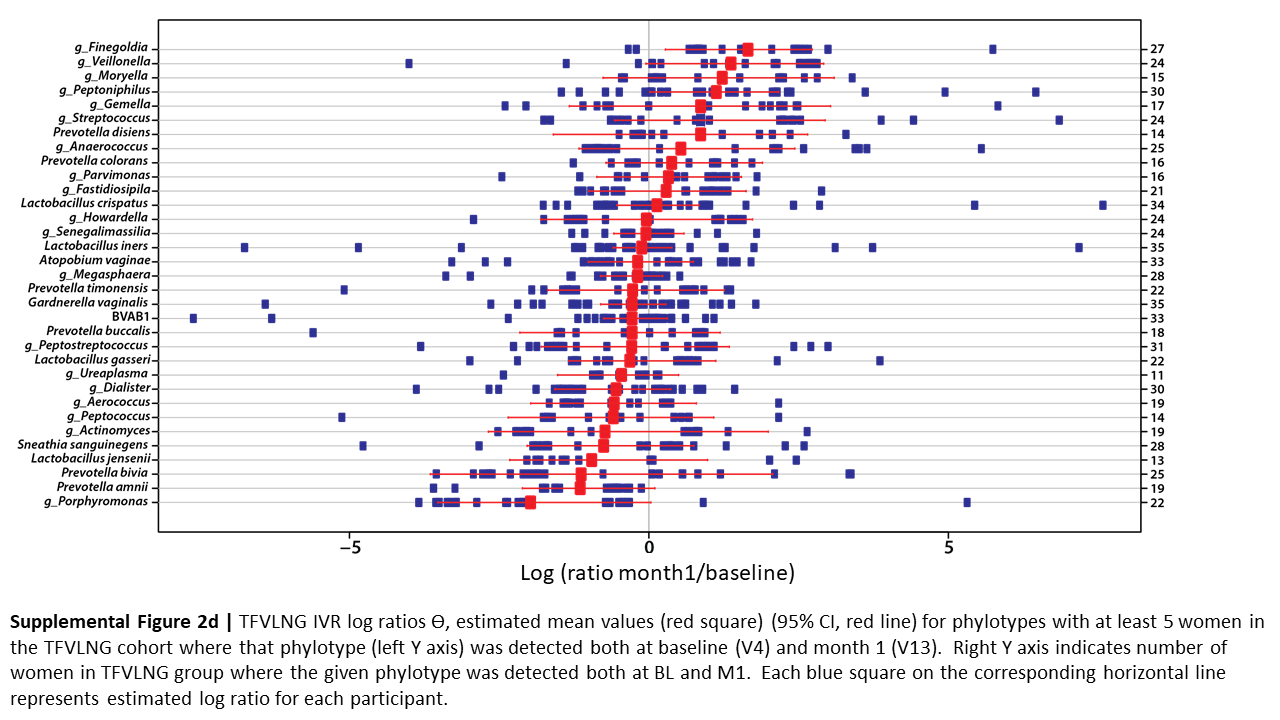

Supplement: Supplementary file 7 [file Image_5.tif]

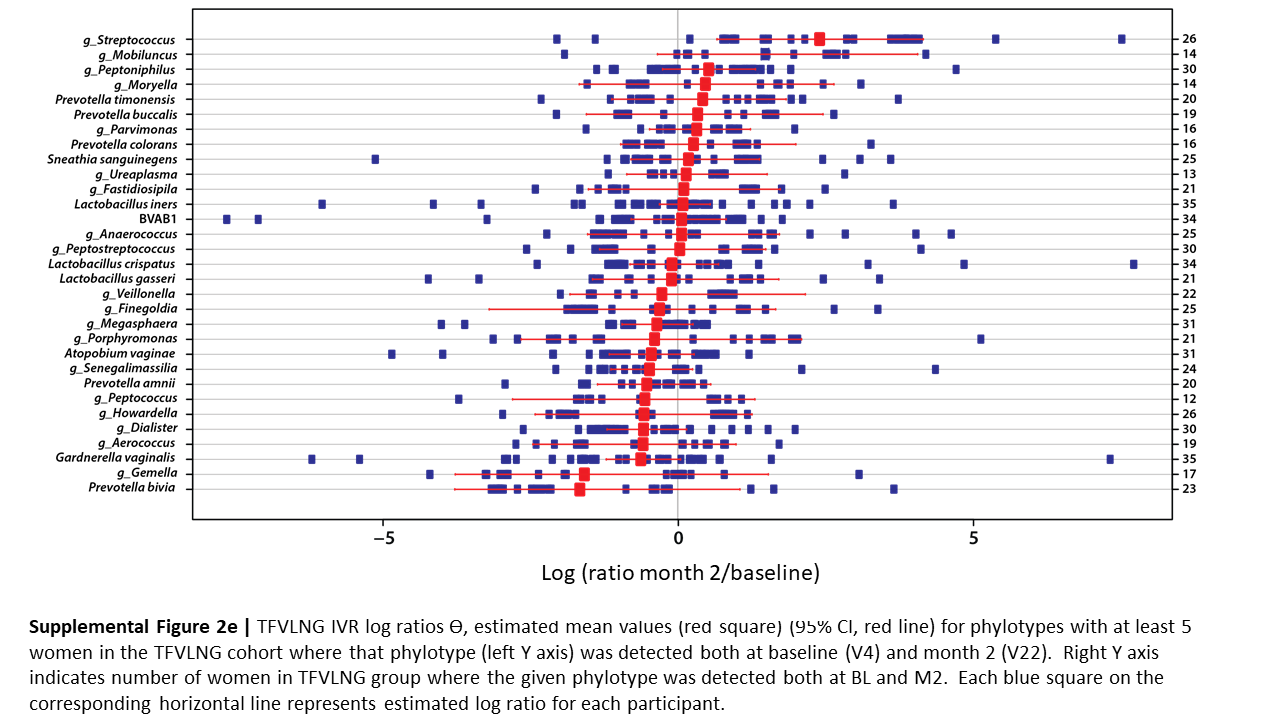

Supplement: Supplementary file 8 [file Image_6.tif]

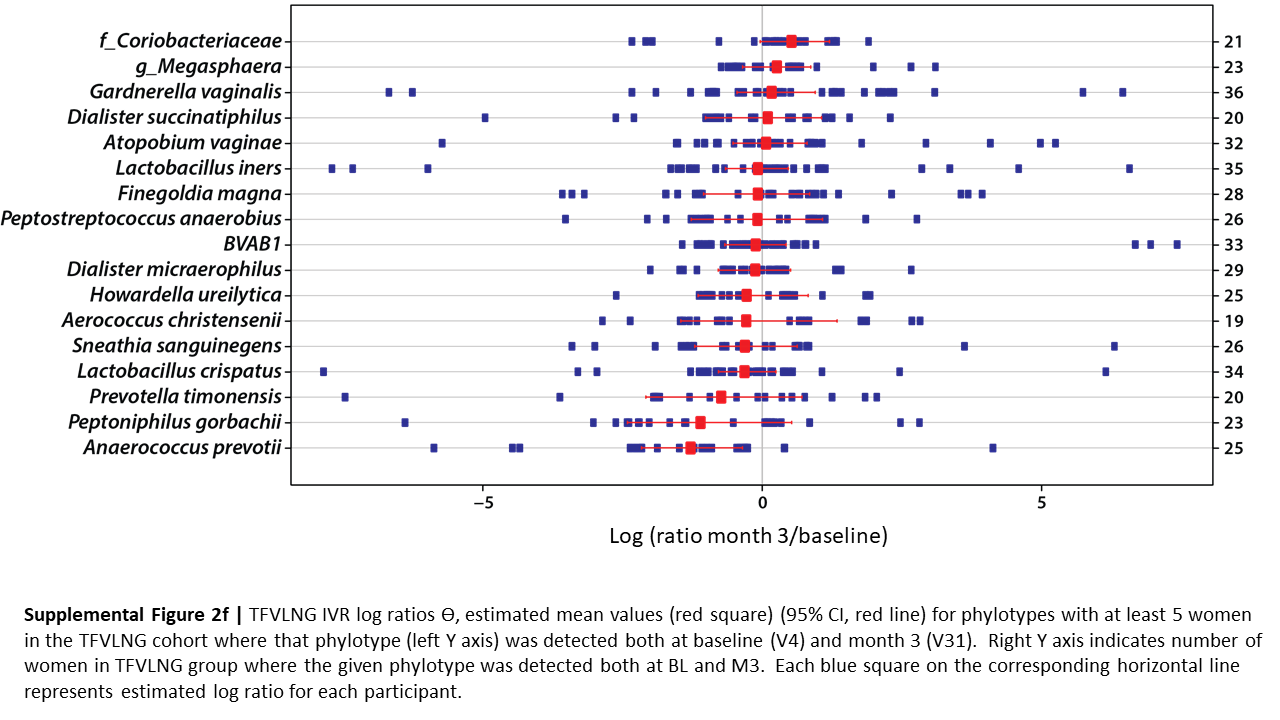

Supplement: Supplementary file 9 [file Image_7.tif]
